# Treatment of Pericardial Effusion Through Subxiphoid Tube Pericardiostomy and Computerized Tomography- or Echocardiography - Guided Percutaneous Catheter Drainage Methods

**DOI:** 10.21470/1678-9741-2018-0077

**Published:** 2019

**Authors:** Abdurrahim Colak, Necip Becit, Ugur Kaya, Munacettin Ceviz, Hikmet Kocak

**Affiliations:** 1 Department of Cardiovascular Surgery, Atatürk University Faculty of Medicine, Erzurum, Turkey.; 2 Department of Vascular Surgery, University Medical Center, Erzurum, Turkey.

**Keywords:** X-Ray Computed Tomography, Pericardial Effusion, Mediastinum, Pericardium, Echocardiography, Retrospective Studies

## Abstract

**Objective:**

In this retrospective study, we aimed to observe the efficacy of pericardial effusion (PE) treatments by a survey conducted at the Department of Cardiovascular Surgery, Faculty of Medicine, Atatürk University.

**Methods:**

In order to get comparable results, the patients with PE were divided into three groups - group A, 480 patients who underwent subxiphoid pericardiostomy; group B, 28 patients who underwent computerized tomography (CT)-guided percutaneous catheter drainage; and group C, 45 patients who underwent echocardiography (ECHO)-guided percutaneous catheter drainage.

**Results:**

In the three groups of patients, the most important symptom and physical sign were dyspnea and tachycardia, respectively. The most common causes of PE were uremic pericarditis in patients who underwent tube pericardiostomy, postoperative PE in patients who underwent CT-guided percutaneous catheter drainage, and cancer-related PE in patients who underwent ECHO-guided percutaneous catheter drainage. In all the patients, relief of symptoms was achieved after surgical intervention. There was no treatment-related mortality in any group of patients. In patients with tuberculous pericarditis, the rates of recurrent PE and/or constrictive pericarditis progress were 2,9% and 2,2% after tube pericardiostomy and ECHO-guided percutaneous catheter drainage, respectively.

**Conclusion:**

Currently, there are many methods to treat PE. The correct treatment method for each patient should be selected according to a very careful analysis of the patient's clinical condition as well as the prospective benefit of surgical intervention.

**Table:** 

Abbreviations, acronyms & symbols				
ANOVA	= Analysis of variance		MRI	= Magnetic resonance imaging
Ca	= Cancer		MVR	= Mitral valve replacement
CRF	= Chronic renal failure		N_2_O	= Nitrous oxide
CT	= Computerized tomography		O_2_	= Oxygen
CVP	= Central venous pressure		PAN	= Polyarteritis nodosa
ECG	= Electrocardiogram		PCR	= Polymerase chain reaction
ECHO	= Echocardiography		PE	= Pericardial effusion
IV	= Intravenous		PPD	= Purified protein derivative
LSD	= Least square difference		SLE	= Systemic lupus erythematosus
MI	= Myocardial injury		SPSS	= Statistical Packet for Social Science

## INTRODUCTION

Pericardial effusion (PE) is the name given to the fluid accumulation in the pericardium leaves. In routine echocardiography (ECHO) controls, this condition occurs in one of every ten patients^[1]^.

The normally existing 15-50 ml of pericardial liquid in humans let the heart work in a frictionless environment. Phospholipids are present in this liquid. The contents of electrolyte and plasma are nearly the same, and the protein content is 1/3 of the plasma. The occurrence of symptoms depends on the amount of liquid, the time of collection, and the physical properties of the pericardium^[1]^.

ECHO is the most valuable diagnostic tool for assessing PE. By using M-mode ECHO, even a small amount of liquid can be detected. The sizes of the effusion are classified considering the anterior and posterior regions of diastolic echo-free space, being lightweight (<10 mm), medium (10-20), and large (> 20 mm)^[2]^. When there is 300 ml of effusion, the separation can be seen both in front and rear regions. In advanced degree of effusion, the image of a pendulum heart is available^[3,4]^. Tomography and magnetic resonance imaging (MRI) can also be used for the detection of PE, and it has been reported that computerized tomography (CT) can be used as a guiding method in treatment of patients with PE^[5]^, especially in cases of postoperative PE. High frequency of localized effusion in postoperative cases makes CT guiding more valuable.

The clinical significance of any PE depends on the presence of an underlying disease and hemodynamic disturbances, determined by the intrapericardial pressure increase. Unless there is evidence of cardiac tamponade and there is no need for pericardiocentesis in small amounts of fluid^[4]^, the underlying cause, if diagnosed (*e.g*., hypothyroidism), should be treated, instead of making the pericardiocentesis. In special conditions, like suspicion of tuberculosis, pericardial biopsy may be required. In this case, subxiphoid pericardiostomy makes it possible, in addition to provide symptomatic relief.

The optimal treatment is controversial. In the absence of an actual tamponade or a high-risk effusion, management should be individualized. In the present study, it was aimed to retrospectively evaluate the results of three drainage techniques: subxiphoid tube pericardiostomy, CT-guided drainage, and ECHO-guided drainage.

## METHODS

Totally, 553 patients were divided into three groups: in the group A, 480 patients underwent subxiphoid pericardiostomy due to PE; in group B, 28 patients underwent CT-guided percutaneous catheter drainage; and in group C, 45 patients underwent ECHO-guided percutaneous catheter drainage. These procedures occurred between 1996 and 2010. Patient's data were obtained from the patients' files. Before 2000, PE, if needed, were treated by subxiphoid drainage. After 2000, in patients with postoperative localized PE, especially in the lateral surface of the right ventricle and posterior surface of the left ventricle, CT-guided drainage has been performed. In patients with general effusion, causing at least 10 mm separation at the right ventricular anterior surface, the ECHO-guided drainage is being preferred.

ECHO was used to determine both the diagnosis and the severity of effusion. While assessing the severity, if the distance between the left ventricular posterior wall and the pericardium during diastole is below 10 mm, it is accepted and classified as mild PE; between 10 mm and 20 mm, it is classified as moderate PE; and over 20 mm, as severe PE^[2]^. Cardiac tamponade was defined by clinical evaluation and ECHO of the patient. Tachycardia, although it is not a lung problem, causes dyspnea and tachypnea and increased the central venous pressure (CVP). In case of presence of classic symptoms, such as hypotension and pulsus paradoxus tamponade, patients were classified as tamponade (Table 1).

**Table 1:** Symptoms of patients with pericardial effusion.

Symptoms	Group A (n=480)	Group B (n=28)	Group C (n=45)
Dyspnea	372 (77.5%)	22 (78.5%)	36 (80%)
Chest pain	218 (45.4%)	13 (46.4%)	27 (60%)
Tachycardia	248 (51.6%)	17 (60.7%)	29 (64.4)
Edema	152 (31.6%)	11 (39.2%)	21 (46.6%)
Fever	123 (25.6%)	8 (28.5%)	11 (24.4%)
Orthopnoea	102 (21.2%)	9 (32.1%)	14 (31.1%)
Abdominal respiration	77 (16%)	4 (14.2%)	8 (17.7%)
Syncope	17 (3.5%)	1 (3.5%)	3 (6.6%)
Cough	116 (24.1%)	11 (39.2%)	19 (42.2%)
Jugular venous distension	228 (47.5%)	13 (46.4%)	22 (48.8%)
Hypotension	48 (10%)	3 (10.7%)	10 (22.2%)

### Subxiphoid Tube Pericardiostomy

The subxiphoid tube pericardiostomy was applied under general anesthesia (n = 30, 6.25%) or local anesthesia supported by sedation (n = 450, 93.75%). General anesthesia was preferred mostly on kids and induction was done with 1,5 mg/kg of ketamine. Also, 0.1 mg/kg of vecuronium neuromuscular block was used and anesthesia was maintained by 60% of nitrous oxide (N_2_O), 40% of oxygen (O_2_), and 0.5-1% of isoflurane.

As there is a hypotension risk in patients who underwent general anesthesia, they were covered and marked with paint before induction. In local anesthesia, 2% lidocaine was injected subcutaneously. Sedation was achieved by the injection of 1 mg/kg of ketamine or 1-2cc of intravenous (IV) midazolam. About 5-6 cm of incision was made from the epigastrium to the xiphoid. After the subcutaneous skin incision, 2% lidocaine was injected into the rectus muscle and the xiphoid again. By cutting the linea alba and the xiphoid and dissecting the subxiphoid tissues, the pericardium anterior face was reached. Then, some amount of liquid was aspirated by the injector; in order to determine whether the hemorrhagic liquid was defibrinated or not, its coagulation was observed. By holding and pulling the pericardium, 3-5 cm² of it was excised for pathological examination. Pericardial fluid samples were collected for cytological, biochemical, and microbiological analyses, then the liquid was poured in a controlled manner; meanwhile, IV digoxin was injected to prevent sudden cardiac dilatation.

Pericardial cavity was visually and manually checked, adhesions and mass lesions were investigated. Adhesions were removed carefully with the help of fingers. With a different incision, 2-4 cm under the former incision, the drainage tube was placed in the pericardial cavity in order to provide postoperative drainage. Making a separate incision from the drainage tube was preferred to prevent the development of postoperative wound infection and incisional hernia. The drainage tube was connected to a closed underwater drainage system, and the subxiphoid incision was closed according to the procedure.

### CT-guided Catheter Drainage Method

The feasibility of performing CT-guided catheter drainage on 28 patients was determined by clinical, echocardiographic, and tomographic assessments.

**Fig. 1:**
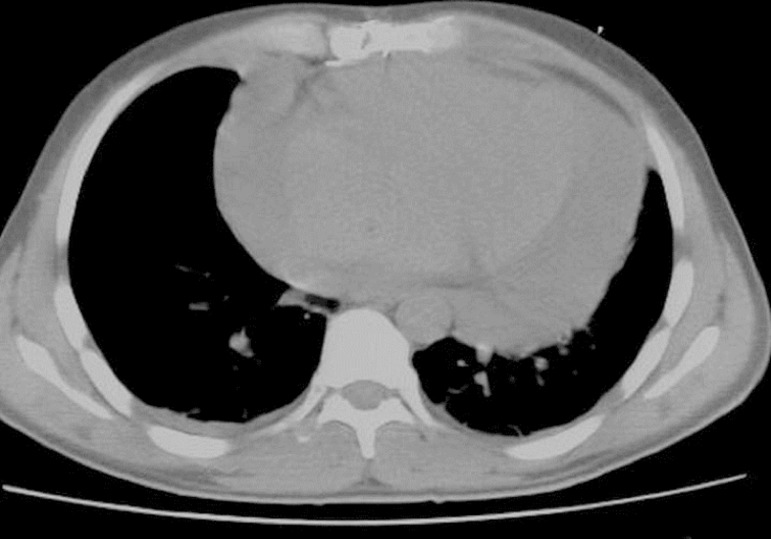
Marking the entry point with computerized tomography (CT).

All patients were assessed by helical CT. In order to determine the entry point of PE drainage, 10 mm range images were taken from the heart apex to the arcus aorta. After the assessment, the skin was marked on the determined entry point (Figure 1). To ensure that the metallic needle was in the right place, CT was performed. And to prevent complications that could occur during electrocardiogram (ECG) monitoring, an intravenous saline with IV opening was inserted, and blood pressure was monitored at frequent intervals.

**Fig. 2:**
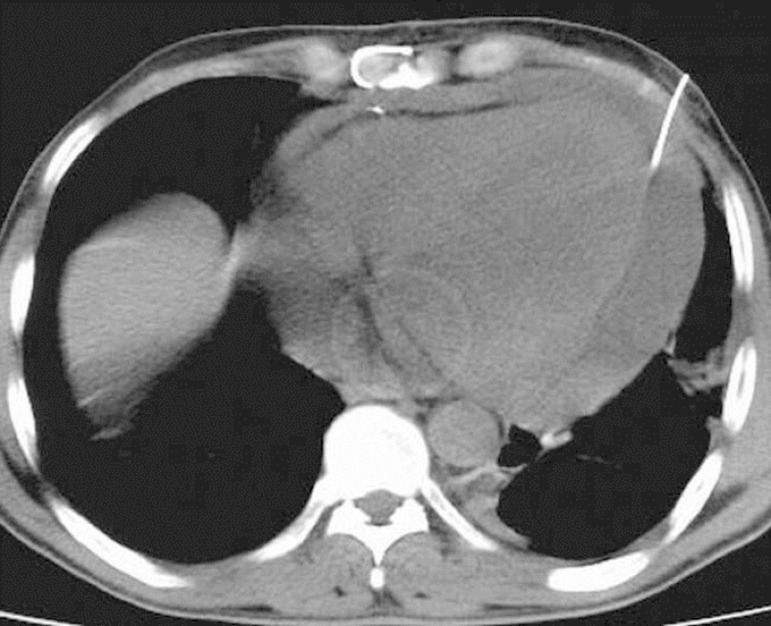
Demonstration of the needle in the pericardial space.

**Fig. 3:**
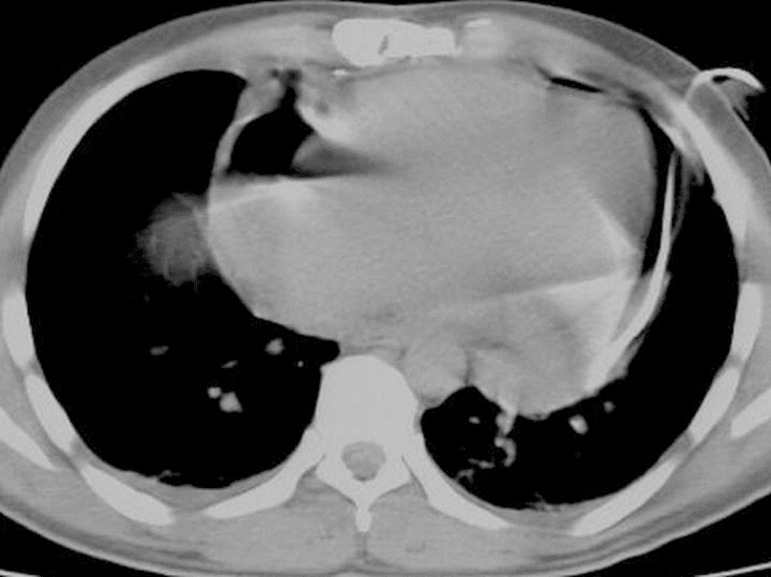
Catheter in the ıntrapericardial area.

**Fig. 4:**
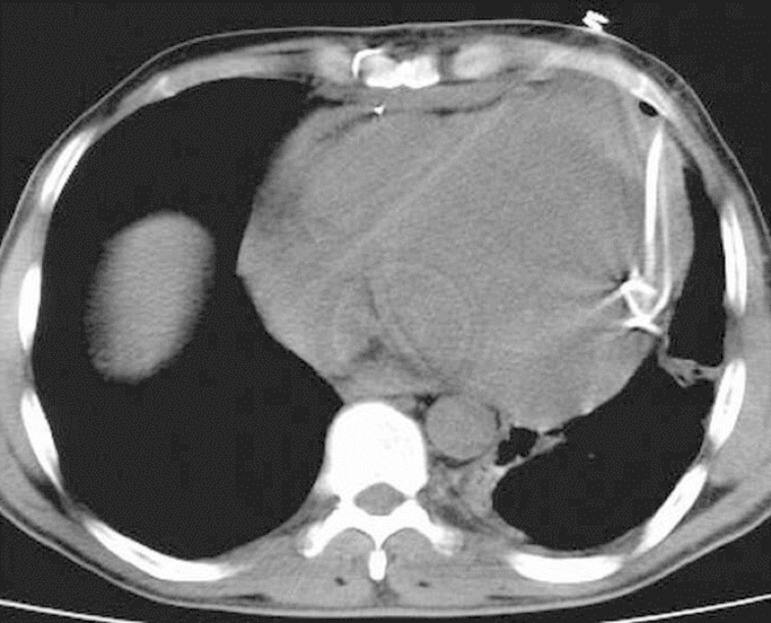
View of reduced intrapericardial liquid.

Having been colored and disinfected, the marked area was covered with sterile drapes. As a local anesthetic agent, 2% lidocaine was used. Then, an 18 G needle with negative puncture was entered to the pericardial cavity, the needle was fixed at the level of liquid aspiration, and the CT was taken again to reaffirm the needle's position (Figure 2). After confirming that it was in the intrapericardial space, the liquid was aspirated and evaluated. If hemorrhagic characteristics were thought to be possible after confirming that the blood defibrinated, a 0.035" guidewire was sent to the pericardial cavity through the needle (Figure 3). The needle was removed and the CT was taken; after confirming that the guidewire was in the intrapericardial cavity, an 8 or 10 F nephrostomy catheter was advanced into the pericardial cavity through a catheter guide. A three-way tap was installed in the tip of the catheter and the liquid was poured into a 50 ml syringe. The catheter was identified and it was connected to the closed underwater drainage system (Figure 4).

### ECHO-guided Pericardiocentesis/Catheter Drainage Method

Having been assessed by ECHO and under intensive care conditions, blood pressure and ECG monitoring were completed, and the patients were operated at the bedside after taking all precautions. After dyeing and disinfecting the subxiphoid area, the patients were put into a 45° tilt position, and they were covered by sterile sheets. After local anesthesia with 2% lidocaine, a small incision was made by an 11" knife. Under the xiphoid, using ECHO as a guide, an 18G needle was lifted, with a slope of 15-20° and negative pressure, aiming at the left shoulder. When the aspiration fluid came, the location of the needle tip was confirmed by ECHO, and an 0038" guidewire was sent to the pericardial space through the needle. After seeing the guidewire in the pericardia via ECHO, a 6F pigtail was sent through guidewire. During this process, the patients' heart rate and blood pressure were frequently measured. The three-way tap was installed at the catheter and fluid was aspirated into a 50 ml syringe. Pericardial liquid was aspirated slowly and intermittently in order to prevent cardiac decompression. Aspiration was done every 6 hours and the amount of liquid was checked by daily ECHO.

### Statistical Analysis

Statistical analyses of the results were done using the Statistical Packet for Social Science (SPSS) 11.0 software and x² and post-hoc tests were applied to the data. In this study, recurrent effusion ratios obtained from the groups, the number of cases developing constriction, complication rates, and 1-month mortality were determined by x² tests, 3x2 contingency table, or 2x2 Fisher's exact test. For the length of in-hospital stay and duration of the catheter or drainage tube stay, a two-way analysis of variance (ANOVA) was used. In bilateral comparisons, *P*-values were obtained by applying the least square difference (LSD) method and interpreted; *P* values < 0.05 were considered significant.

## RESULTS

A total of 553 patients were included in the study. The mean age was 6 ± 79 years-old, and 25 of them were children.

### Subxiphoid Tube Pericardiostomy

Data belonging to 480 patients who underwent subxiphoid tube pericardiostomy - ages ranging from 6 months to 84 years (mean 35.7 years), 261 of whom being men (55%) and 219 being women (45%) - were examined. ECHO detected mild PE in 16 patients (3%), medium PE in 193 patients (40%), and severe PE in 271 patients (57%). All of the patients with traumatic PE had tamponade symptoms (n = 25). Some patients also underwent invasive procedures like blunt thoracic trauma, coronary angioplasty, stent implantation, and temporary endocardial pacemaker implantation (n = 20).

In all patients with symptomatic PE, relief was provided by subxiphoid tube pericardiostomy. Intraoperative myocardial injury (MI) occurred in five patients (1%). In these cases, upon failing to control bleeding with subxiphoid approach, urgent median sternotomy was done. In two patients, bleeding occurred during the removal of adhesions connected to the tuberculous pericarditis; in other three cases, bleeding occurred during the placement of the tube connected to the traumatic rupture of the right atrium. None of the patients were lost.

The reasons for the subxiphoid tube pericardiostomy in the patients were: uremic pericarditis (n = 208, 43.3%), idiopathic and unidentified pericarditis (n = 92, 19.1%), malignancies (n = 65, 13.5%), tuberculous pericarditis (n = 50, 10.4%), nontuberculous bacterial pericarditis (n = 24.5%), trauma (n = 25, 5.2%), rheumatoid arthritis (five), systemic lupus erythematosus (SLE) (five), hypothyroid (four), and polyarteritis nodosa (PAN) (two) (Table 2).

**Table 2:** Causes of pericardial effusion.

Cause of pericardial effusion	Group A	Group B	Group C
Uremic pericarditis	208 (43.3%)	2 (7.1%)	10 (22.2%)
Idiopathic pericarditis	92 (19.1%)	10 (35.7%)	-
Tuberculosis pericarditis	50 (10.4%)	-	7 (15.5%)
Bacterial pericarditis	24 (5%)	1 (3.5%)	8 (17.7%)
Trauma (operation, angioplasty comp. )	25 (5.2%)	13 (46.4%)	4 (8.8%)
Malignancy	65 (13.5%)	2 (7.1%)	13 (28.8%)
Others	16 (3.3%)	-	3 (6.6%)
Total	480	28	45

During the operation, the amount of drainage was 50-4500 ml (average 1258±720) and the average amount of drainage during the postoperative periods was 980 ± 255 ml. The approximate duration of postoperative drainage was 4.3 ± 1.6 days. The maximum drainage was from a uremic pericarditis patient, and the minimum drainage was from a 6-month-old baby with bacterial pericarditis. The fluid was transudate in 228 cases (47.5%), hemorrhagic in 138 cases (28.7%), and exudate in 85 cases (6%).

Forty-seven (72%) of the 65 PE patients had malignancies, and malignancy cells were positive in the investigation of pericardial material and/or liquid. Twenty-eight of the 65 patients had lung cancer (Ca), 16 had lymphoma, 12 had breast Ca, seven had leukemia, and two had malignant thymoma.

Twenty-two of the 50 patients with tuberculous pericarditis had positive preoperative purified protein derivative (PPD) and microbiological laboratory test results. Through cytological examination of pericardial fluid taken during surgery, 45 (90%) patients were diagnosed with tuberculous pericarditis. Five of eight patients having negative polymerase chain reaction (PCR) were diagnosed with tuberculous pericarditis in cytological examination of pericardial fluid. Preoperative diagnosis of tuberculosis in three patients could not be confirmed in the received materials and examinations; however, they continued their treatment as they were accepted as tuberculosis patients.

The microorganisms obtained from fluid sample cultures of patients with pericarditis were Mycobacterium tuberculosis (n = 40), Pneumococcus (n = 11), Viridans streptococci (n = 7), Haemophilus influenzae (n = 4), and Staphylococcus (n = 3).

Patients stayed at the hospital between three and 30 days (average 3 ± 27 days). Wound infection was seen in 27 (5.6%) patients. Most of these patients were female and obese. There was not any intraoperative mortality. During the hospital stay, seven of 480 patients (1.4%) died. Four of those patients had a diagnosis of congestive heart failure, and despite inotropic therapy in the postoperative period, they died because of low cardiac output and multiorgan failure.

Within the first postoperative 30 days, there was a need of additional surgery for PE in 45 patients (9.3%). Twenty-four of the recurrent PE patients had uremic pericarditis, 15 had tuberculous pericarditis, three had idiopathic pericarditis, and three were sticked to malignancy. Recurrence rate in patients with tuberculosis was found to be 30% in 15 patients.

In all of the patients with effusion recurrence, incision was made from the epigastrium, left to the sternocostal junction and a pericardiopleural window was opened. None of the patients with pericardiopleural window developed recurrence at one-year follow-up. All patients were followed up for one year. After 6-12 months follow-up due to the development of malignant effusion, three patients had the pericardiapleural window opened through a left anterior mini-thoracotomy. Two of these patients had tuberculous pericarditis, and one had uremic pericarditis due to effusion. After the operation, no recurrence happened during the follow-up (Tables 2 and 3).

**Table 3:** Comparison of the length of in-hospital stay and duration of the drainage tube stay between the  groups.

	Group	Group	Mean	*P*
Hospitalization time	A	B	-103.199	<0.05
		C	-88.938	<0.05
	B	A	103.199	<0.05
		C	14.262	0.064
	C	A	88.938	<0.05
		B	-14.262	0.064
Drainage tube time	A	B	-13.327	<0.05
		C	-15.431	<0.05
	B	A	13.327	<0.05
		C	-0.2103	0.634
	C	A	15.431	<0.05
		B	0.2103	0.634

### CT-guided Catheter Drainage Method

Among the 28 patients who underwent PE drainage with CT-guided catheter drainage method, 15 were female (53.5%) and 13 were male (46.5%). Their ages varied between 1 and 80 years (mean 41.3 years). The most important symptom in these patients was dyspnea. Relief was observed in the symptoms of 25 patients who were successfully operated. ECHO detected moderate effusion in ten of these patients (35.7%) and severe effusion in 18 (64.3%). Thirteen of these patients (46.4%) had previously undergone open-heart surgery. These patients had effusions, two of them due to malignancy, one due to bacterial infection, and two due to uremia. Other ten patients (35.7%) had idiopathic PE (Table 2).

The effusion localization was in the left ventricular lateral in 14 patients, left ventricular posterior in eight patients, right atrium posterolateral in five patients, and right ventricular posterior in one patient. In the patients whose daily amount of drainage were followed, it was applied three-way tap for the aspiration of liquid when necessary. When the daily amount of drainage was < 50 ml, catheters were taken guided by ECHO on the lack of intrapericardial liquid.

As for complications, upon development of hemopericardium, depending on the pericardial laceration, subxiphoid tube pericardiostomy was performed under local anesthesia in one patient. Although the needle entered the intrapericardial space in one patient, the liquid was not aspirated. So, considering that there could be organized hematoma under general anesthesia, the hematoma was evacuated by opening a pericardial window through anterior mini-thoracotomy.

In one case in which was applied CT-guided catheter drainage method due to uremic PE, ECHO and CT detected the existence of fluid and the presence of catheter in the thorax; because it was a right pleural effusion in severe level, pericardiopleural window was opened with right mini-thoracotomy.

About 1000 ml of liquid was evacuated from thorax and 1000 ml was from the pericardial cavity. In the remaining 25 patients (93%), the desired results were obtained from the operations performed.

In 11 patients (39%), it was aspirated serous fluid; in 10 patients (38%), hemorrhagic fluid; in seven patients (25%), transudate fluid; and in one patient, purulent fluid. The amount of aspirated fluid was 50-1500 ml (mean 920 ± 225). The minimum drainage was from chronic renal failure (CRF) patients; the maximum drainage was from an idiopathic pericarditis patient.

In two (7.1%) of the patients who underwent CT-guided catheter drainage, there was recurrence; again, the catheter drainage was applied with the same method and no recurrence was observed. There were no mortalities due to the process. One patient died three days after the surgery because of fulminant hepatitis. After being discharged from hospital, one patient who had a mitral valve replacement (MVR) died in the intensive care unit because of overdose of coumadin due to cerebral hemorrhage (Tables 3 and 4).

**Table 4:** Comparison of the results between the groups.

	Group A	Group B	Group C	*P*
Recurrent effusion	45 (9.4%)	2 (7.1%)	7 (15.6%)	0.365
Construction	14 (2.9%)	-	1 (2.2%)	0.638
Complication	5 (1%)	1 (3.5%)	1 (2.2%)	0.425
Drainage time	3-15 (4.3±1.6)	2-17 (5.6±3.6)	2-15 (5.9±2.6)	<0.05
Hospitalization time	3-30 (5.57)	7-30 (15.89)	7-30 (14.46)	<0.05
Mortality (first month)	7 (1.4%)	1 (3.5%)	1 (2.2%)	0.655

### ECHO-guided Pericardiocentesis/Catheter Drainage Method

ECHO-guided pericardiocentesis was performed in 45 patients; 28 of them were males (62.2%) and 17 were females (37.8%). Their ages ranged from 19 to 76 years (mean 42.7 years). In these patients, the most common symptom was dyspnea. In ten patients (22.2%), there was overt tamponade.

Fifteen patients (33.3%) had moderate effusion, and 30 (66.7%) had severe effusion. Ten of these patients (22.2%) had CRF, seven (15.5%) had tuberculous pericarditis, 13 (28.8%) had malignancy, four (9%) had iatrogenic complications of angiography, eight (17.7%) had infection, and three (6.6%) had effusion related to acute MI. Pericardiocentesis was performed successfully in all patients. The average number of punctions were 1.3 ± 0.6. In all patients, clinical relief was observed after the procedure and their symptoms decreased. In one patient, acute left heart failure developed after the procedure and it was cured by medical treatment.

No major complication occurred during the procedure, except for one patient in whom subcutaneous hematoma occurred by puncture to subcutaneous vascular structure; the hematoma was regressed by applying ice. In this patient, hematocrit fall did not occur.

200-1500 ml (average 1278 ± 620) of liquid were discharged from the patients. Hemorrhagic fluid was aspirated from 18 (40%) patients, serous fluid from 27 (60%) patients. Six of the seven patients with tuberculous pericarditis were identified as PCR positive. In one patient, the adequate identification could not be done by liquid cytology and microbiology. Then, subxiphoid tube pericardiostomy was performed in four of these patients. In the culture of infective pericarditis of eight patients, Streptococcus viridans and Staphylococcus were detected.

The average hospital stay of these patients was 7-30 days. No mortality occurred during this period. The catheter was extracted in an average of 5.9 ± 2.6 days. In seven patients (15.5%), after the catheter was extracted, subxiphoid pericardiostomy was needed due to recurrence of PE; no re-recurrences were observed in these patients later. Four of these patients had tuberculous pericarditis, two had uremic pericarditis, and one had PE due to malignancy. During the one-year follow-up, seven patients died from infarction: five malignancy patients, due to malignancy; one patient with CRF, due to the primary disease; and one patient with MI. On the development of constrictive pericarditis in one patient with tuberculous pericarditis, surgical pericardiectomy was performed (Tables 3 and 4).

## DISCUSSION

Many diseases that can cause pericarditis and virtually any disease that can involve the pericardium can cause PE. In this study, in which the causes of PE were presented, those were often associated with underlying causes, as uremia, malignancy (lung, breast, ovarian carcinoma, and lymphoma), many bacterial infections, few viral infections, myocardial infarction, and autoimmune diseases. In most studies, uremic pericarditis is a rare cause of PE, however, in ours, it emerged as the most common cause^[4]^. Since our hospital is the dialysis center of the region, the incidence of uremic PE is high.

The most important step in management of PE is to determine if tamponade is present or if there are any features suggesting a high chance of developing tamponade in the near time. While the tamponade rate was 44% in previous studies, 27% of the patients had clinical tamponade in our study^[5,6]^.

Symptomatic PE can be treated by various procedures. Pericardiocentesis with CT- or ECHO-guided catheter drainage, subxiphoid tube pericardiostomy, and subxiphoid or anterior thoracotomy with pericardiopleural window opening can be used in the treatment of PE. Among these methods, the most effective one must be chosen according to the patient's clinic and history. Therefore, the optimal treatment modality is controversial^[7-9]^. In the absence of an actual tamponade or a high-risk effusion, management should be individualized.

Cardiac pericardiocentesis in tamponade patients is a life-saving method. However, more can be done safely in 10 mm and over effusions during diastole. The most common and serious complication of pericardiocentesis is the laceration and perforation of myocardium. ECHO- or scope-guided methods will reduce the risk^[10]^. There were not such complications in our studies. During pericardiocentesis, serious arrhythmias, vascular hemorrhages, pneumothorax, infections, and major vagal reactions have also been reported^[11,12]^. As Park et al.^[13]^ have noted in the summary, they have shown that PE can be monitored by videothoracoscopy under local anesthesia. In a study by Palma et al.^[14]^, videothoracoscopy has shown excellent mediastinal and chest inspection ability, and it may be performed on a safe and fast manner, besides providing elements that could change the diagnosis and, consequently, the specific treatment of some patients.

In traumatic pericarditis and especially in purulent pericarditis, surgical drainage is more preferred^[13,15]^. In our study, in the patients who underwent subxiphoid tube pericardiostomy, the complication rate with CT and ECHO guidance was lower than in patients who underwent catheter drainage (1%).

The ideal procedure should be easily implemented, should result in minimal morbidity and mortality, should provide full and permanent drainage, should not be recurrent, and should provide material for adequate histological, cytological, and microbiological analyses to identify the cause of the effusion^[10,16]^. All the treatments were successful and have been sufficient to ensure drainage of symptomatic effusion. CT- or ECHO-guided catheter drainage, subxiphoid tube pericardiostomy, and anterior mini-thoracotomy with pericardiopleural window opening were the drainage methods in our clinic. The advantages of catheter drainage with CT or transthoracic ECHO guidance were no need for incision and general anesthesia, and it was less painful. These methods are less successful, and the risk of complications is high in minimal effusion and posteriorly located effusions. Pericardial biopsy samples cannot be taken with catheter methods. The advantages of subxiphoid tube pericardiostomy are that it allows the visualization of the pericarditis and pericardial cavity of the pole and that it makes possible a complete drainage and to get pericardial tissue for pathological examination. In our studies, from the 480 PE patients who underwent subxiphoid tube pericardiostomy, 462 (96.2 %) were subjected to local anesthesia with sedation and 28 (5.8%) to general anesthesia. General anesthesia is usually preferred in children. Local anesthesia was applied to patients in whom we performed catheter drainage with transthoracic ECHO and CT guidance.

Although pericardiocentesis is life-saving in instable patients, pericardial biopsy samples cannot be extracted; also, pericardiocentesis is inadequate to diagnose tuberculosis in purulent pericarditis and invasive malignant cases^[17,18]^. Although this method ensures some relief in patients with symptoms of tamponade, it is not suitable for definitive therapy^[17,18]^. Even in the presence of active tuberculous pericarditis, culture taken from pericardial liquid may be negative. Therefore, in addition to the pericardial fluid drainage, it is recommended to make a pericardial biopsy^[10]^. Since the tuberculous pericarditis' treatment is long and difficult, the risk of recurrence and constriction of this disease is higher than of other diseases^[18-20]^. In our study, patients with tuberculosis pericarditis were the most prone to develop constrictive pericarditis. The low sociocultural level of the patients and their non-adherence to treatment have led to the development of recurrent effusions and constriction^[19]^.

Malignancy is another condition leading to cardiac tamponade. In centers where there are many oncology patients, most of those have malignancies; in other centers, benign pathologies may occur more frequently^[21]^. In the acutely symptomatic patient, pericardiocentesis provides immediate relief of symptoms but it is associated with high recurrence rates. Patients with a limited expected lifespan can be managed with repeated pericardiocentesis or extended pericardial catheter drainage. For patients with a long life expectancy, surgical drainage provides the highest freedom from recurrence^[22]^. In a multicenter study by Moores et al.^[23]^ with 155 patients, the rate of tamponade due to malignancy was 53% and the rate of tamponade due to tuberculosis was 2%. In our study, the rate of tamponade due to tuberculosis was 13.5% in group A patients, 7.1% in group B patients, and 28.8% in group C patients. In PE due to malignancy, the treatment method should be selected by examining the patient's condition. Although there are intrapericardial treatment methods, there are no randomized studies providing their reliability. Recurrence was observed in approximately 50-70% of the malignant PE patients^[17]^. For these reasons, subxiphoid pericardiostomy, a very small portion using pericardiopleural window, was often performed in our patients. Allen et al.^[17]^ performed subxiphoid tube pericardiostomy in 94 of the 117 patients with cardiac tamponade; 23 hemodynamically stable patients underwent percutaneous catheter drainage. 0% of mortality and one complication (1.1%) were observed in patients with subxiphoid tubes pericardiostomy. While 17% of complications were reported in patients who underwent percutaneous drainage, only 4% them showed mortality. Recurrence was reported as 1.1% in patients who underwent subxiphoid tube pericardiostomy and as 30% in those who underwent percutaneous drainage. In cases of tamponade with PE, percutaneous drainage was reported in those who didn't have hemodynamic stability. It was ensured that subxiphoid tube pericardiostomy was more effective and trustworthy in those who had hemodynamic stability.

In our study, as there was apparent tamponade in 10 (22.2%) cases, we've performed catheter drainage with echocardiographic pericardiocentesis at bedside and the relief of symptoms was observed. In addition, in 15 of the patients who underwent ECHO-guided catheter drainage because of the recurrence of PE, a subxiphoid tube pericardiostomy was performed and no recurrence was observed later. None of these patients developed major complications, except one patient who developed hematoma because of subcutaneous arterial puncture; the hematoma went down with the application of cold. No fall was observed in the patients' hematocrit levels. Mortality was 0% in patients who underwent subxiphoid tube drainage. While no mortality was seen due to operations, patients died from primary diseases. Recurrence in these patients was 9.4%, and most of them were tuberculous pericarditis patients.

Cegielski et al.^[19]^ identified PCR positive in 14 of the 20 tuberculous pericarditis patients. In this study, 42 of the 50 patients who underwent subxiphoid tube pericardiostomy were reported as PCR positive. In our studies, we concluded that subxiphoid tube pericardiostomy is more valuable as it allows biopsy for diagnosis and treatment.

Palatianos et al.^[24]^ reported that microorganisms developed in seven of the eight exudative PE patients. In our study, there was a breeding in the pericardial fluid samples that were extracted from the 65 patients who underwent subxiphoid tube pericardiostomy and in four patients who underwent ECHO-guided catheter drainage.

Data obtained from our patients showed the development of recurrent effusion (*P*=0.365), constriction rate (*P*=0.638), complication rate (*P*=0.425), and mortality rate in the first follow-up month (*P*=0.655). There was no significant difference between the three groups (*P*<0.05) (Table 2).

There are several notable limitations in this study. This is a retrospective study that is subject to be studied in detail and subject to selection bias; nevertheless, propensity matching was utilized to partially account for these biases. Other reasons for limitations are that this study was performed at a single center, that may not reflect the patients' characteristics and etiologies seen at other institutions, and that long-term follow-up was not possible in many of our patients, limiting our analysis to short-term outcomes.

## CONCLUSION

As a conclusion, pericardiocentesis can be always necessary for diagnosis of the cause of PE. Although there are many methods of treatment of PE, which method should be used in which patient must be selected by careful examination, the patient's clinic results, and the treatment effectiveness. Although 2-D ECHO is the diagnostic imaging modality of choice for the initial evaluation of PE, CT can be important when more precise localization and quantification of pericardial fluid are necessary, when an effusion is complex or loculated, or when a clot is present. We believe that CT-guided catheter drainage method should be preferred when the patient's comfort is at the forefront, when there is no need to take the diagnostic tissue sample, and in postoperative PE cases. To overcome the intrinsic limitation of the pericardiocentesis technique, it is recommended to perform the procedure with image guidance whenever it is possible. Especially in patients with tamponade, ECHO-guided catheter drainage is an outstanding treatment method that can be done bedside to provide emergency relief. However, as there is no possibility of biopsy, we believe that it can be inadequate in terms of diagnostic value because it allows only the examination of the liquid. Subxiphoid tube pericardiostomy is a more effective and trustworthy method; it is thought that this method is more effective in diagnosis and treatment as it allows biopsy, examination of the pericardial space, and removal of the adhesions. Further studies should be carried out at multi-centered conditions to verify the results.

**Table:** 

Authors' roles & responsibilities	
AC	Conception and design of the work; revising it critically for important intellectual content; final approval of the version to be published
NB	Conception and design of the work; revising it critically for important intellectual content; final approval of the version to be published
UK	Conception and design of the work; revising it critically for important intellectual content; final approval of the version to be published
MC	Conception and design of the work; revising it critically for important intellectual content; final approval of the version to be published
HK	Conception and design of the work; revising it critically for important intellectual content; final approval of the version to be published
